# Effect of Different Milk Diet on the Level of Fecal Calprotectin in Very Preterm Infants

**DOI:** 10.3389/fped.2020.00552

**Published:** 2020-09-16

**Authors:** Simonetta Costa, Maria Letizia Patti, Alessandro Perri, Carmen Cocca, Giovanni Pinna, Chiara Tirone, Milena Tana, Alessandra Lio, Giovanni Vento

**Affiliations:** Department of Woman and Child Health and Public Health, Child Health Area, Fondazione Policlinico Universitario A. Gemelli, IRCCS, Università Cattolica del Sacro Cuore, Rome, Italy

**Keywords:** fecal calprotectin, inflammation, immunomodulation, milk diet, preterm infants

## Abstract

**Objective:** To evaluate the course of fecal calprotectin (FC) in very preterm infants over the first 15 days of life in relation to the type of milk diet.

**Methods:** This study was part of a randomized controlled trial comparing two different ways of integrating the own mother's milk (OMM) for the evaluation of feeding tolerance in very preterm infants. In infants with gestational age of ≤ 32 weeks randomized to receive preterm formula (PF group) or pasteurized donor human milk (PDHM group) as a supplement to the OMM insufficient or unavailable, FC level was planned to be measured at the first meconium passage and at days 8 and 15 of life (T0, T1, and T2, respectively).

**Results:** FC data were available for all the 70 infants randomized, 35 in the PF group, and 35 in the PDHM group. The mean FC levels were similar in the two study groups at T0 and T1, whereas they were significantly higher in the PF group than the PDHM group at T2. FC values decreased over the first week of life in both groups and significantly increased over the second week of life only in the PF group.

**Conclusions:** Our study demonstrates a significant increase in FC levels when PF is used as a supplement to the OMM compared to the use of PDHM. Further studies are needed to establish if the higher FC levels in infants receiving PF are the expression of a normal immunological maturation rather than an initial inflammatory process.

## Introduction

Calprotectin is a 36.4-KDa calcium and zinc-binding protein that constitutes the main component of cytosolic protein of neutrophils, monocytes, and macrophages. Calprotectin was shown to have bactericidal and fungicidal properties, and it may be involved in the regulation of inflammation (1, 2). Calprotectin is found in various body fluids in proportion to the degree of inflammation, but its concentration in the stool is ~6 times that of the plasma: this is the reason why the measurement of fecal calprotectin (FC) would likely be a sensitive and specific marker of gastrointestinal inflammation. The level of FC can be proportional to the quantity of neutrophils migrating through the gastrointestinal mucosa (3, 4).

In adults and children, FC is greatly used as a noninvasive marker for the diagnosis of inflammatory bowel disease and may serve as an important clinic test in order to evaluate the inflammatory state of the whole intestinal tract (5, 6). The reference value given for healthy adults and children older than 4 years of age is 50 μg/g of feces (7).

The current evidence suggests that FC is elevated in newborn infants suffering from necrotizing enterocolitis, but its significance as an early screening marker remains unknown. The reported sensitivity and specificity of the test remain unidentified, as there is a lack of consensus regarding the implementation of specific cutoff values (8).

Conflicting results have also been reported on the effect of the kind of feeding on the level of FC in infants. Some studies have shown higher FC in exclusively breastfed infants when compared to mixed- or formula-fed ones, and some have shown no difference (9–20).

The aim of this study was to evaluate the time course of FC in very preterm infants over the first 15 days of life in relation to the different kind of milk diet.

## Materials and Methods

This study was part of a single-center, randomized, non-inferiority trial comparing two different ways of integrating the own mother's milk (OMM) over the first 2 weeks of life for the evaluation of feeding tolerance in very preterm infants (21). Briefly, the clinical trial planned that infants with gestational age (GA) of ≤ 32 weeks, without congenital malformations, connatal infections, or abnormal antenatal Doppler flow velocimetry, and for whom OMM was unavailable or insufficient to satisfy the planned enteral intakes, were randomized to receive pasteurized donor human milk (PDHM) or preterm formula (PF) as a supplement to the OMM. The PF diet consisted of 3.5 g of protein/100 Kcal formula (Plasmon PreZero, Plasmon, Italy). Donor human milk came from mothers who prematurely delivered and pasteurized within 24 h of collection by the Holder method (+ 62.5°C for 30 min). Minimal enteral feeding was initiated within the first 48 h of life and was continued at 20 ml/kg/day for up to 5 days. Subsequently, enteral nutrition volume was increased by 20 ml/kg/day. The parenteral nutrition (PN) was started soon after birth in all infants with a birth weight (BW) of <1,250 g and in infants with BW of <1,500 g requiring invasive ventilation. The PN was stopped when enteral intake was >125 ml/kg/day.

FC level was measured at the first meconium passage and at days 8 and 15 of life (T0, T1, and T2, respectively).

Stool samples were taken from the infants' diapers with sterile plastic spoons and put in sterile plastic screw-cap tubes. The samples were then frozen at −80°C until analysis. One hundred milligrams of stool were weighed, placed in a 15-ml conical tube, and agitated with a wooden stirrer. An extraction buffer was added, the sample vortexed to form a fine slurry, and then placed on a shaker for 25 min. One milliliter was removed and centrifuged at 10,000 g for 20 min. The supernatant was removed for analysis by ELISA (Eurospital, Trieste, Italy). The FC was expressed as μg/g of stool. Every assay included a standard curve and quality controls, all samples were done in duplicate, and the intra-assay coefficients of variation were <10%.

All parameters were checked for normal distribution using the Shapiro–Wilk test. Differences between the two groups were investigated using paired Student's *t*-test for normally distributed data or Wilcoxon signed-rank test otherwise. Chi-square test was used to compare categorical data. Data are presented as mean ± standard deviation (SD) or median–interquartile range. A *p*-value of <0.05 is considered to indicate statistical significance. Statistical analysis was performed using XLSTAT version 2014.5.03.

The institutional review boards approved the study, and written informed consent was obtained from the parents of all subjects before enrollment.

## Results

FC data were available for all the 70 preterm infants randomized in the original trial, 35 in the PF group and 35 in the PDHM group. Demographic and clinical data were well balanced between the two study groups, as detailed in Table 1.

**Table 1:** Demographic and clinical data.

	**PF**	**PDHM**	***p***
	***N* = 35**	***N* = 35**	
Gestational age, weeks	30.2 ± 1.7	30 ± 1.9	0.73
Birth weight, g	1,342 ± 275	1,365 ± 332	0.75
Male gender, *n*	18 (51)	14 (40)	0.47
Small for gestational age, *n*	4 (11.4)	4 (11.4)	1
Antenatal steroids, *n*	29 (83)	30 (86)	1
Sepsis, *n*	2 (5.7)	5 (14.3)	0.42
NEC, *n*	0	0	1
RDS, *n*	17 (48.6)	23 (65.7)	0.22
PDA, *n*	10 (28.6)	8 (22.8)	0.78
BPD, *n*	1 (2.8)	1 (2.8)	1
Length of hospital stay, days	37.5 ± 17.9	39.3 ± 18.6	0.68
Mortality, *n*	0	1 (2.9)	1

*Data are presented as number (percentage) or mean ± SD. PF, preterm formula; PDHM, pasteurized donor human milk; NEC, necrotizing enterocolitis; RDS, respiratory distress syndrome; PDA, patent ductus arteriosus; BPD, bronchopulmonary dysplasia*.

There was no difference in the maximum weight loss, but infants in the PF group regained birth weight 2 days earlier than infants in the PDHM group. No difference was observed in the duration of fasting, minimal enteral feeding, and parenteral nutrition. Time to reach full enteral feeding of 150 ml/kg/day of milk was similar in the two study groups. Infants in both groups received a similar total intravenous and enteral fluid intake, such as a similar amount of OMM, whereas the amounts of PF and PDHM were significantly different between groups, as expected (Table 2).

**Table 2:** Nutritional data and milk intakes over the first 15 days of life.

	**PF**	**PDHM**	***p***
	***N* = 35**	***N* = 35**	
Maximum weight loss, %	12.6 ± 4.5	11.9 ± 5.1	0.55
Time to regain birth weight, days	12.9 ± 2.6	15.0 ± 5	0.032
*Nihil per os*, days	2.0 ± 1.7	1.7 ± 1.7	0.57
Minimal enteral feeding, days	3.0 ± 2.6	2.6 ± 2.2	0.52
Time to full enteral feeding, days	12.3 ± 7.0	12.8 ± 6.5	0.76
Parenteral nutrition duration, days	9.1 ± 6.8	7.9 ± 6.6	0.45
Total fluids intake, ml/kg/day	142.3 ± 6.3	143.6 ± 7.7	0.45
Total intravenous fluids intake, ml/kg/day	70.6 ± 42.7	64.3 ± 41	0.52
Total enteral fluids intake, ml/kg/day	68.2 ± 41.1	76.5 ± 37.6	0.29
OMM, ml/kg/day	19.1 ± 24.5	23.7 ± 27.6	0.45
OMM, % of total enteral intake	28	31	1
PDHM, ml/kg/day	0.0 ± 0.0	52.8 ± 37.5	<0.001
PF, ml/kg/day	49.1 ± 42.7	0.0 ± 0.0	<0.001

*Data are presented as mean ± SD or percentage. OMM, own mother's milk; PF, preterm formula; PDHM, pasteurized donor human milk*.

The mean FC levels were similar in the two study groups at T0 and T1, whereas they were significantly higher in the PF group than the PDHM at time 2 (Table 3).

**Table 3:** Fecal calprotectin levels over the study period.

	**PF**	**PDHM**	***p***
T0 F-Calprotectin (μg/g)	214 ± 166	203 ± 152	0.88
T1 F-Calprotectin (μg/g)	123 ± 117	124 ± 116	0.82
T2 F-Calprotectin (μg/g)	182 ± 138	119 ± 116	0.05

*Data are presented as mean ± SD. T0, first meconium passage; T1, day 8 of life; T2, day 15 of life; PF, preterm formula; PDHM, pasteurized donor human milk*.

**Figure 1:**
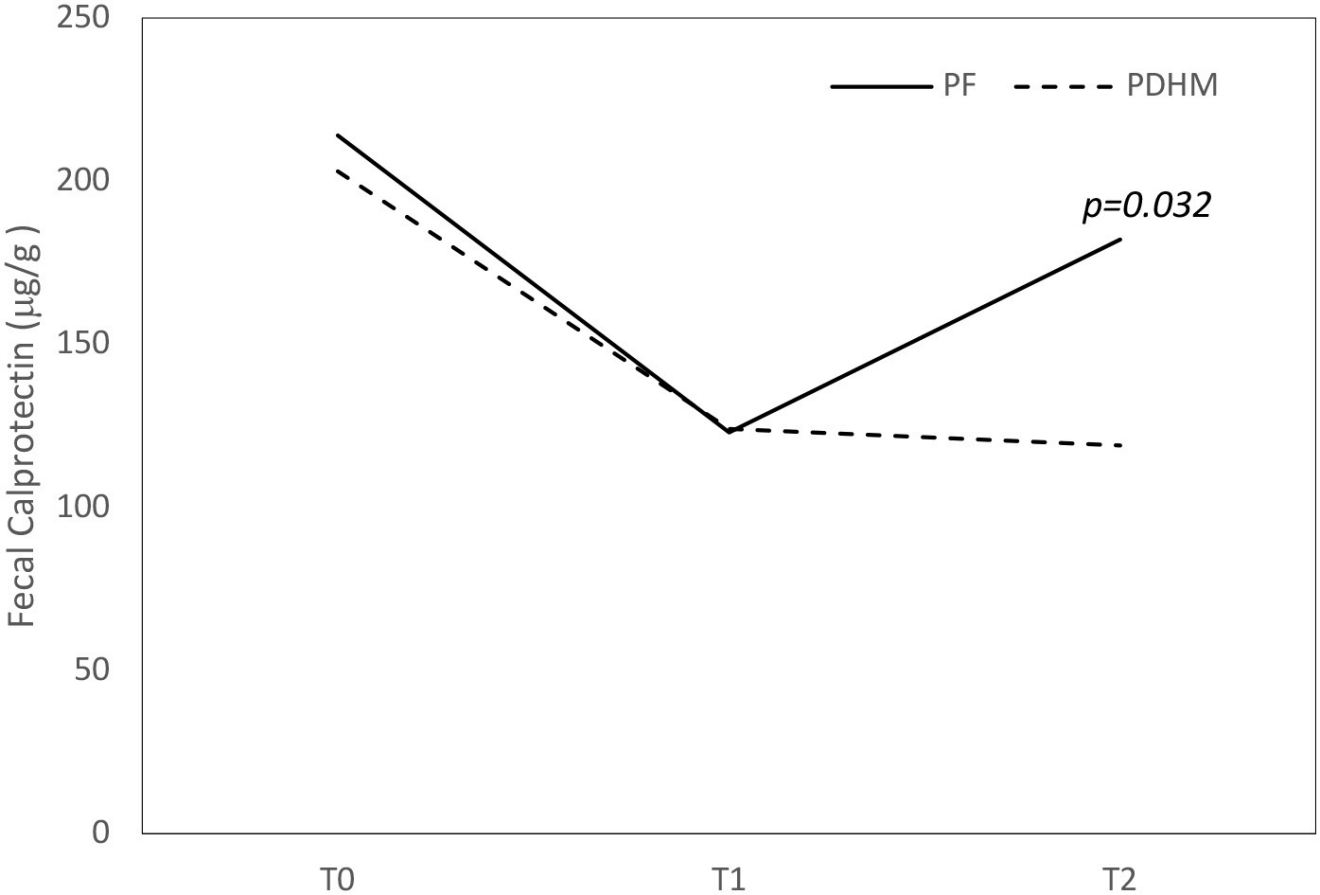
Trend of fecal calprotectin values over the first 15 days of life. The figure shows that fecal calprotectin concentration decreased over the first week of life in both groups and increased at 15 days only in the PF feed group. PF, preterm formula; PDHM, pasteurized donor human milk.

FC values decreased over the first week of life in both groups and increased in a significant manner in the second week of life only in the PF group (Figure 1).

## Discussion

In this study, we observed significantly higher FC levels at 15 days of life in very preterm infants who received PF as integration to OMM compared to those infants who received PDHM.

Previous studies reported conflicting results on the effect of feeding on the FC levels in infants since some authors observed higher FC levels in breastfed infants compared to mixed- or formula-fed ones (9–13), while others authors found no difference in the FC levels of infants fed breast milk vs. formula (14–20).

It is a challenge to explain why FC levels are higher in breastfed infants than formula-fed ones since FC is an index of bowel inflammation. On the contrary, it is evident that human milk contains several anti-inflammatory bioactive components that promote intestinal growth and maturation and reduce intestinal permeability more than formulas (22–25). It has been hypothesized that higher FC in breastfed neonates could be consistent with the promotion of maturation of the intestinal mucosa. High FC in the gut, induced by human milk, could provide a protective effect through its bactericidal and fungicidal properties and could contribute to modulation of the intestinal microflora (10, 11, 13).

This hypothesis seems to do not support our results as we found higher FC levels in the PF group compared to the PDHM group. We supposed that the significantly higher FC values in formula-fed infants at the 15th day of life could be explained with a subclinical inflammation of the bowel induced by PF.

It has been reported that preterm infants with feeding intolerance have statistically significant elevated levels of FC than those without feeding intolerance (26, 27) and that the FC levels decrease in a significant manner when PF-fed infants with feeding intolerance were shifted to receive amino acid-based formula (27). In our original trial, the infants who received PF as integration to OMM had a similar feeding tolerance than those who integrated their OMM with PDHM, since the time to achieve the full enteral feeding was the same for both groups (21), so we cannot attribute the higher FC levels in preterm infants receiving PF to the feeding intolerance.

A significant positive correlation has also been observed between the amount of volume of enteral feeding and FC levels (17, 28), suggesting that exposure to the increasing luminal dietary antigens might induce a state of physiological subclinical intestinal inflammation and then an increase in the calprotectin in the gut lumen. In our study, infants in both groups received the same mean enteral fluid intake, but FC increased significantly at 15 days of life only in the PF group.

It is almost evident that the type of milk diet influences the diversity of the gut microbiota in infants. The greatest microbial richness has been found in infants exclusively fed OMM (29). Cong et al. (30) found a more favorable microbial community in infants receiving exclusively OMM and a significantly lower diversity and a different microbial community in infants receiving PHDM and PF compared to infants receiving OMM. In particular, these authors found that infants receiving OMM had the highest abundance of Clostridiales, Lactobacillales, and Bacillales and the lowest abundance of Enterobacteriales. The groups of infants receiving OMM+PHDM as well as those infants receiving OMM+PF also had higher abundance of Clostridiales, Lactobacillales, Bacillales, and Bacteroidales compared to the groups of infants receiving only PHDM or PF or PHDM+PF (30).

The microbiota related to OMM stimulates the normal development of the gut-associated lymphoid tissue, representing the major external driving force in the maturation of the immune system after birth. When the gut microbiota becomes established, neutrophil infiltration into the intestinal mucosa occurs as an integral part of normal bowel development. This physiological inflammatory process would explain why infants who receive breast milk have higher levels of FC. In our study, infants in both groups received a similar amount of OMM, and the variable that can explain the difference in FC levels is attributable to the PF.

In our study, it is difficult to establish if the higher levels of FC found in PF group infants are the expression of a physiological process of intestinal maturation rather than the beginning of a pathological inflammatory process. However, the levels of FC we found were below the lowest reported cutoffs for bowel pathology in preterm infants (31, 32), as well as feeding tolerance was similar in both groups.

Regarding the course of FC over the first 15 days of life, we observed that the FC concentration decreased over the first week of life in both groups and increased at 15 days only in the PF-fed group. The course of FC in PF group infants is consistent with that reported by Zoppelli et al. (33), who found that in preterm infants, the FC levels, initially high in the meconium, dropped, reaching a nadir at approximately 8 days of life and then increased in a GA-dependent manner except in the most premature infants with GA <26 weeks. We could speculate that the failure to increase the FC levels in PDHM group infants might be interpreted as an inappropriate immune response of the bowel as what happens in extremely premature babies probably because the donor human milk, after the pasteurization process, is unable to induce the same maturation process induced by the raw OMM.

Our study has some strengths but also limitations. The strength of our study is that it is part of a randomized controlled trial and therefore there is a good control of all the variables that could influence the levels of FC. A limitation of the study is not having studied the intestinal microbiota which could have given more information about the significance of FC levels in relation to the integration of OMM with PF.

Our study demonstrates a significant increase in FC levels when PF is used as a supplement to the OMM when insufficient or unavailable compared to the use of PDHM.

Further studies are needed to establish whether this increased FC levels is the expression of a normal immunological maturation process rather than an initial inflammatory process related to the use of PF.

## Data Availability Statement

The raw data supporting the conclusions of this article will be made available by the authors, without undue reservation.

## Ethics Statement

The studies involving human participants were reviewed and approved by Institutional Review Board, Department of Woman and Child Health and Public Health, Child Health Area, Fondazione Policlinico Universitario A. Gemelli, IRCCS; Università Cattolica del Sacro Cuore. Written informed consent to participate in this study was provided by the participants' legal guardian/next of kin.

## Author Contributions

All authors have read and approved the manuscript for submission have made a substantial contribution to the conception, design, gathering of data and a contribution to the writing and intellectual content of the article and acknowledge that they have exercised due care in ensuring the integrity of the work. SC conceived the design of the study, interpreted the data, and revised it critically for intellectual content. MP drafted the article and interpreted the data. AP analyzed the data for the work. CC, GP, CT, MT, and AL acquired the data and researched the scientific literature. All authors revised the article and have given final approval of the version to be published. GV final approved the work to be published.

## Conflict of Interest

The authors declare that the research was conducted in the absence of any commercial or financial relationships that could be construed as a potential conflict of interest.
